# Procrastination among basic science undergraduate medical students in a Caribbean medical school

**DOI:** 10.15694/mep.2017.000055

**Published:** 2017-03-17

**Authors:** Pathiyil Ravi Shankar, Surekha M. Bhat, Neelam R. Dwivedi, Atanu Nandy, Byron Barton

**Affiliations:** 1 Xavier University School of Medicine

**Keywords:** Academic procrastination, Caribbean, medical, prevalence, self-reported

## Abstract

This article was migrated. The article was marked as recommended.

Purpose: The study was conducted to study procrastination behavior among basic science undergraduate medical students using the previously validated procrastination assessment scale students (PASS). Frequency of and reasons for procrastination were compared among different subgroups of respondents.

Methods: The study was conducted during the first two weeks of February 2017 using PASS. Gender, nationality and semester of study of the respondents were noted. PASS explores areas of and frequency of procrastination, reasons for procrastination and interest in changing the behavior. The frequency of procrastination, fear of failure, risk aversiveness, laziness and rebellion against control scores were compared among different subgroups using appropriate statistical tests.

Results: A total of 107 students (84.9%) participated in the study. The mean frequency of procrastination score was 32.9 (maximum score 60). The score was significantly correlated with the respondents’ gender. With regard to the percentage of students who nearly always or always procrastinated on a task, the percentages with regard to completing assignments, studying for exams, completing reading assignments, academic administrative tasks, attendance tasks and school activities in general were 25.2, 19.7, 25.2, 19.6, 18.7 and 17.7. The mean score for ‘fear of failure’ and ‘aversiveness of task’ as described by Solomon and Rothblum was 2.29 and 2.83. The mean scores for fear of failure, risk taking, and laziness were 26.17, 13.76 and 14.32. The median rebellion against control score was 6. Risk taking score was higher among respondents of other nationalities compared to Americans.

Conclusions: Procrastination was regarded as a greater problem with regard to studying for exams and completing reading assignments and preparing for problem-based learning sessions. Only 42% of students were interested in attending a program to overcome procrastination. Similar studies among students during the clinical years are required. A study correlating self-reported procrastination with behavior can be carried out. Procrastination can also be studied in other offshore, Caribbean medical schools.

## Introduction

In medical schools and other academic settings, students have a number of tasks to perform ranging from participating in various activities including small group sessions, attending clinics, completing reading and writing assignments, studying for exams and completing different academic administrative and attendance tasks (Steel, 2004). The general tendency to delay participating in and completing these tasks is termed academic procrastination. Procrastination has been defined as ‘A purposive, habitual, intentional and needless delay in beginning or completing tasks, which prevents individuals from reaching their goals’ ( Lay, 1986). Strong and consistent predictors of procrastination were task aversiveness, task delay, self-efficacy, impulsiveness, and conscientiousness among others. The prevalence of procrastination has been noted to be different in various studies ranging from 15 to 46% ( Ferrari et al., 2009; Ferrari, 2001).

Long-term procrastination may lead to negative outcomes such as anxiety, stress, and depression ( Scher & Ferrari, 2000). Different factors leading to academic procrastination have been mentioned in the literature. Among these are fear of failure, perfectionism, task aversion, rebellion against control, and risk taking ( Ozer & Ferrari, 2011; Onwuegbuzie & Collins, 2001). Studies have shown that academic procrastination is associated with impaired academic achievement ( Lakshminarayan et al., 2013; Madhan et al., 2012). Identifying procrastination among medical students and the underlying reasons behind this behavior is important so that the extent of the problem is understood and remedial measures carried out where required.

One of the instruments to study procrastination among students is the procrastination assessment scale students (PASS) which was developed by Solomon and Rothblum ( Solomon & Rothblum, 1984). The different components of the questionnaire are frequency of procrastination, reasons for procrastination and interest in changing procrastination behavior. PASS has been used in a number of studies conducted in different settings ( Mortazavi et al., 2015; Ozer et al., 2009; Rothblum et al., 1986; Howell & Watson, 2007).

Xavier University School of Medicine (XUSOM) is an offshore, Caribbean medical school admitting students from the United States (US), Canada and other countries to the undergraduate medical (MD) course. Students spend six semesters learning the basic sciences in Aruba and then complete their clinical rotations in affiliated hospitals in the US and Canada. The basic sciences are taught in an integrated, organ system-based manner with early clinical exposure ( Shankar, 2015a). A semester of study at the institution is of 15 weeks duration and there are three student intakes a year in January, May, and September. Procrastination has not been previously studied among basic science students at the institution. Hence, the present study was conducted to obtain information about frequency of procrastination, reasons for and interest in changing procrastination behavior. The different scores were compared among different subgroups of respondents using appropriate statistical tests and the level of correlation between these scores and respondents’ demographic characteristics was also noted.

## Methods

The study was conducted among undergraduate basic science medical students (first, second, third, fifth and sixth semester) at the institution during the first and second weeks of February 2017. The fourth semester was not offered during the Spring 2017 semester. The previously used and validated procrastination assessment scale for students (PASS) was used for the study after obtaining written permission from the developer/s. Students were explained the aims and objectives of the study and invited to participate. Written, informed consent was obtained from all participants. The study was approved by the institutional review board of the institution vide notification XUSOM/IRB/2017/01.

The gender, nationality and semester of study of the respondents were noted. The instrument dealt with three areas related to procrastination. These were frequency of procrastination, reasons for procrastination and respondent interest in changing their procrastination behavior. The section on areas and frequency of procrastination had a set of 18 questions which respondents answered according to a Likert scale scored from 1 to 5. A frequency of procrastination score was obtained by adding together the scores of statements 1, 2, 4, 5, 7, 8, 10, 11, 13, 14, 16 and 17. The tasks which were studied were completing assignments, studying for exams, keeping up with weekly reading assignments and preparing for problem-based learning (PBL) and other sessions, academic administrative tasks, attendance tasks and school activities in general. The normality of distribution of the frequency of procrastination score was tested using the one sample Kolmogorov-Smirnov (KS) test (p <0.05). The frequency of procrastination scores was compared among different subgroups of respondents using appropriate statistical tests. The correlation between frequency of procrastination and the respondents’ demographic characteristics was studied at a 5% level of significance. Statements 3, 6, 9, 12, 15 and 18 measured respondents’ willingness to decrease their tendency to procrastinate on a particular task.

The second section of the questionnaire studied respondents’ reasons for procrastination. Respondents rated their reasons for procrastination for each individual statement according to a five-point Likert scale ranging from not at all reflects why I procrastinated to definitely reflects why I procrastinated. The fear of failure subscale was calculated as the mean of items 19, 24, 33, 39, and 42 while the aversiveness of task subscale was calculated as the mean of items 27, 34, and 35. These scores were calculated as recommended by Solomon and Rothblum ( Solomon & Rothblum, 1984). These scores were compared among different subgroups of respondents using appropriate statistical tests. The correlation between fear of failure score and different respondent demographic characteristics was calculated at a 5% level of significance. A similar correlation was also conducted for aversiveness of task. The risk taking subscale was the mean of items 30 and 36 while the rebellion against control subscale was the mean of items 25 and 38. Item 21 dealt with difficulty in making decisions, and item 42 was termed as dependency. The normality of distribution of the different subscales and of individual items was tested using the one sample KS test (p <0.05). the average scores of the different individual items were calculated.

A study conducted in Turkey ( Ozer et al., 2009) had derived a different set of factors with regard to the parameter ‘Reasons for procrastination’. These were fear of failure, risk-taking, laziness and rebellion against control. As many of our students though of US or Canadian nationality were of Asian origin we also calculated the scores according to the framework recommended by Ozer et al. Fear of failure (FF) was calculated by summing up statements 19, 21, 23, 24, 26, 28, 29, 31, 33, 41 and 42. Risk taking (RT) was calculated by adding statements 20, 30, 32, 36, 37, 40 and 44. Laziness (L) was obtained by adding the scores of statements 22, 27, 34, 35 and 43 while rebellion against control (RC) was the sum of statements 25, 38, and 39. The one sample KS test was used to compare the normality of distribution of these variables. The average scores were compared among different subgroups of respondents using appropriate statistical tests.

Respondents’ answers to different questions regarding their interest in changing their procrastination behavior were described using number and percentage. This was the focus of the third section of the questionnaire.

## Results

One hundred and seven of the total of 126 students (84.9%) participated in the study and completed the questionnaire. Table 1 shows the demographic details of the respondents. The mean frequency of procrastination score was 32.90 (maximum possible score 60). The score was found to follow a normal distribution and hence mean and standard deviation were used as the measures of central tendency and dispersion. The frequency of procrastination score was significantly correlated with the respondents’ gender (P= 0.023). There was no significant correlation with other demographic characteristics. The scores were compared among dichotomous subgroups using independent samples t-test while analysis of variance was used for others. With regard to the percentage of students who nearly always or always procrastinated on a task, the percentages with regard to completing assignments, studying for exams, completing reading assignments, academic administrative tasks, attendance tasks and school activities in general were 25.2, 19.7, 25.2, 19.6, 18.7 and 17.7. With regard to the percentage of students who mentioned that procrastination on a task was nearly always or always a problem for them, the percentage for completing assignments, studying for exams, completing reading assignments, academic administrative tasks, attendance tasks and school activities in general were 19.7, 31.7, 25.2, 14, 15 and 12.1.

The median score of students with regard to reducing their tendency to procrastinate on completing assignments was 4, indicating a degree greater than somewhat but lower than definite. The median score for reducing the tendency to procrastinate while studying for exams was 5 indicating a definite desire. The median score for reducing the tendency to procrastinate with regard to preparing for PBL and keeping up with their weekly reading assignments was 4. The scores for reducing the tendency to procrastinate with regard to academic administrative tasks, attendance tasks and school activities in general was 3 (somewhat) indicating a lower priority.

The mean ± SD score for ‘fear of failure’ as a reason for procrastination was 2.29 ± 0.99 (maximum possible score being 5) while the mean ± SD score for ‘aversiveness of task’ was 2.83 ± 0.99 (maximum possible score being 5). These scores were noted to follow a normal distribution and hence mean and standard deviation were used as the measure of central tendency and variation. The scores were compared among dichotomous subgroups using independent samples t-test while analysis of variance was used for others. There was no significant correlation with different respondent demographic characteristics for these two scores. The mean ± SD score for rebellion against control was 2.10 ± 1.18 (maximum possible score being 5). The mean score for risk taking was 1.94 (maximum score being 5). The mean score for difficulty making decisions, lack of assertion and dependency were 2.81, 2.19 and 2.44 respectively.

The factors relating to reasons for procrastination were also scored as described by Ozer and coworkers ( Ozer et al., 2009). The factors FF, RT and F were noted to follow a normal distribution while the distribution of RC was not normal. The mean ± SD scores for FF, RT and L scores were 26.17 ± 7.99 (maximum score 50), 13.76 ± 5.43 (maximum score 35) and 14.32 ± 4.63 (maximum score 30). The median RC score was 6 (maximum score 15). The scores were not significantly different among male and female respondents. The risk taking behavior was significantly higher among respondents of other nationalities compared to students of American nationality. Fear of failure score was significantly higher among the third semester students compared to the fifth semester. No other significant differences in scores among different subgroups was noted. Table 4 shows the individual responses to different statements regarding respondents’ interest in changing their procrastination behavior.

## Discussion

The mean frequency of procrastination score in the present study was 32.90 (maximum score being 60). The score was higher among male students and the score had a significant correlation with the gender of the respondents. The mean ± SD score for ‘fear of failure’ as a reason for procrastination was 2.29 ± 0.99 (maximum possible score being 5) while the mean ± SD score for ‘aversiveness of task’ was 2.83 ± 0.99 (maximum possible score being 5). The mean frequency of procrastination score in the present study was comparable to that reported by Solomon & Rothblum ( Solomon & Rothblum, 1984). Their scores were 32.61 for students who had incorrectly marked their ID numbers and 33.53 for the group who correctly provided their ID numbers. In general, the frequency of self-reported procrastination and the degree to which procrastination on a task was regarded as a problem by the respondents was lower than that reported by Solomon & Rothblum ( Solomon & Rothblum, 1984). In a study conducted in Turkey, the mean frequency of procrastination score was 36.8 which is higher than that reported presently. In Turkey ( Ozer & Ferrari, 2011), students procrastinated more when studying for exams, reading assignments and completing term papers (30%) than that reported in the present study. The degree of procrastination for academic administrative, attendance and general school activities was however, higher in the present study.

Procrastination was higher among male students in the present study. A similar finding was noted in the Turkish study ( Ozer & Ferrari, 2011). The reasons for greater procrastination among male students reported in a separate study were fear of failure, laziness, risk taking and rebellion against authority ( Ozer et al., 2009). In the present study there was no significant difference in the frequency of procrastination among different semesters of basic science students. A similar result was reported in the Turkish study ( Ozer et al., 2009).

The average FF, RT, L and RC scores were compared with those noted in the Turkish study ( Ozer et al., 2009). The mean FF, RT and L scores were higher in the Turkish study. The median RC score seems to be higher in the present study when compared with the mean score reported in Turkey. The Turkish study was conducted among undergraduate students enrolled in different departments of a reputable public university while the present study was done among undergraduate basic science medical students in a private medical school. Hence it is difficult to directly compare these findings.

The authors of the Turkish study reported that female students procrastinate more on their academic tasks due to fear of failure and laziness as compared to male students. A similar result was noted in another study ( Senecal et al., 1997). The Turkish study authors partly explained this citing the conservative nature of Turkish society. Archer and Lloyd had mentioned that women may tend to be more fearful and avoidant as a result of hormonal constitution ( Archer & Lloyd, 1982). Solomon and Rothblum also reported that female students were more likely to endorse items which dealt with fear of failure when compared to males ( Solomon & Rothblum, 1984).

Nearly 58% of students were not interested in attending a program which focused on overcoming procrastination. This may be partly related to the lower levels of self-reported procrastination in the present study. Most respondents wanted less than 5 sessions in total and one session a week, Fifty-seven percent of respondents wanted the sessions to be offered on weekdays and nearly 50% wanted a group size of less than 20 students. A large percentage were interested in either using a group discussion format or a combination of formats for the sessions. Small group, active learning strategies are widely used in the institution and students are generally in favor of these learning methods ( Shankar, 2015b).

The high response rate was the strength of the present study. Responses were obtained using a standardized instrument, PASS which has been used in a variety of settings. The study also had limitations. Not all respondents completed all the required statements/questions. Students during the clinical years were excluded due to logistic challenges. PASS is a self-report instrument and it is possible students may have underreported the extent of and the reasons for procrastination behavior. We did not correlate self-reported procrastination with behavior as had been done previously.

## Conclusions

The frequency of procrastination score was lower than that reported in the literature. The score was higher among males but was not significantly different among different semesters or nationalities. Reasons for procrastination were also studied. Procrastination was regarded as a greater problem with regard to studying for exams and completing reading assignments and preparing for PBL sessions. Only 42% of students were interested in attending a program to overcome procrastination. Similar studies among the students during the clinical years are required. A study correlating self-reported procrastination with behavior can be carried out. Procrastination can also be studied in other offshore, Caribbean medical schools.

## Take Home Messages

• . The present study examined self-reported procrastination behavior among basic science undergradute medical students at a Caribbean medical school.
• The frequency of procrastination score was lower than that reported in the literature and was higher among males.
• Procrastination was regarded as a greater problem with regard to studying for exams and completing reading assignments and preparing for PBL sessions
• Only 42% of students were interested in attending a program to overcome procrastination
• Studies among students during the clinical years are required.

## Notes On Contributors

P Ravi Shankar is Professor of Medical Education and Professor of Pharmacology at the Xavier University School of Medicine, Aruba.

Surekha M Bhat is Professor of Biochemistry at the Xavier University School of Medicine, Aruba.

Neelam R Dwivedi is Professor of Introduction to Clinical Medicine and Director of the Standardized Patient program at the Xavier University School of Medicine, Aruba.

Atanu Nandy is Professor of Microbiology at the Xavier University School of Medicine, Aruba.

Byron Barton is Professor of Histology and Dean of Premedical Sciences at the Xavier University School of Medicine, Aruba.

## Appendices

**Table 1.** Demographic characteristics of respondents

Characteristic		Number (percentage) ^*^
Gender	Male Female	54 (50.5) 49 (45.8)
Nationality	United States Canadian Others	47 (43.9) 30 (28.0) 27 (25.2)
Semester	First Second Third Fifth Sixth	28 (26.2) 14 (13.1) 35 (32.7) 15 (14.0) 15 (14.0)

^*^ The numbers may not add up to 107 and the percentages to 100 in all instances as some respondents did not fill in all the demographic characteristics

**Table 2.** Frequency of procrastination scores among different subgroups of respondents

Characteristic	Mean score	P value
Gender		
Male Female	34.52 30.75	0.071
Nationality		
US Canadian Others	32.17 32.5 34.52	0.707
Semester		
First Second Third Fifth Sixth	32.68 32.00 32.88 33.00 34.07	0.978

**Table 3.** Fear of failure and aversiveness of task scores among different subgroups of respondents

Characteristic	Fear of failure		Aversiveness of task	
	Mean score	P value	Mean score	P value
Gender				
Male Female	2.29 2.33	0.617	2.78 2.80	0.933
Nationality				
US Canadian Others	2.12 2.29 2.54	0.322	2.57 3.09 3.02	0.075
Semester				
First Second Third Fifth Sixth	2.28 2.17 2.69 1.72 2.08	0.617	2.52 2.86 2.98 2.60 3.22	0.933

**Table 4.** Interest in changing procrastination behavior among the respondents

Statement	Response	Number (percentage)
Would you be interested in attending a program that focuses on overcoming procrastination if such a program were offered next semester?	Yes No	44 (41.1) 62 (57.9)
How many program sessions in total would you be willing to attend if a procrastination program were offered?	None < 5 5-10 >10	34 (31.8) 50 (46.7) 16 (15.0) 6 (5.6)
How many sessions per week would you be willing to attend?	None One Two Three	35 (32.7) 45 (42.1) 21 (19.6) 5 (4.7)
What time would be the best for you in scheduling such a program?	None Morning Lunchtime Afternoon Evening	32 (29.9) 25 (23.4) 17 (15.9) 21 (19.6) 11 (10.3)
What days would be the best for you in scheduling such a program?	No days Weekdays Weekends	32 (29.9) 61 (57.0) 12 (11.2)
How large a group would you prefer?	Not interested <10 10-20 Group size does not matter	35 (32.7) 30 (28.0) 24 (22.4) 15 (14.9)
I feel that a program to improve procrastination habits would be:	Unnecessary Somewhat useful Extremely useful Useful, but not for me	19 (17.8) 47 (43.9) 26 (24.3) 12 (11.2)
What format would be most interesting to you?	Not interested Group discussion Lecture Following a written manual Combination	32 (29.9) 31 (29.0) 12 (11.2) 2 (1.9) 29 (27.1)
