# Supplementary material for: Procrastination among basic science undergraduate medical students in a Caribbean medical school
Source: MedEdPublish (2016). 2017 Mar 17;6:55. [Version 1] doi: 10.15694/mep.2017.000055 (PMC10885246; doi:10.15694/mep.2017.000055)
Supplement: Peer review history

# Reviewer report: Reviewer response for version 1

**DOI:** 10.21956/mep.19456.r28670

**Published:** 2018-07-20

**Authors:** Sateesh Babu Arja

**Affiliations:** 1 Avalon University School of Medicine

This review has been migrated. The reviewer awarded 4 stars out of 5 I thoroughly enjoyed reading this paper. I must congratulate the authors for taking up such a cumbersome procedure and study at a Caribbean medical school which are generally a low-resource context with limited availability of the faculty time. Very well written and thoroughly explained the methods used. But I would like to see more about the backgorund of the students including the admission criteria and the tools was used in the selection of medical students. It is interesting to see that 57.9% of students said no to attending a program that focuses on overcoming procrastination if such a program were offered in the next semester. Some of these students answered differently for the rest of the questions in the same category. Finally, it would have been better if there are any conclusions drawn and remedial procedures to correct the procrastination rather than just saying that the current study score was better than previous studies.

Reviewer Expertise:

NA

No decision status is available

# Reviewer report: Reviewer response for version 1

**DOI:** 10.21956/mep.19456.r28668

**Published:** 2017-04-03

**Authors:** Sandra Kemp

**Affiliations:** 1 Curtin Medical School, Curtin University

This review has been migrated. The reviewer awarded 3 stars out of 5 I think this study deals with an interesting topic. It is useful when a study attempts to administer an existing instrument (in this case, the Procrastination Assessment Scale - Students) in a context different to the one in which the instrument was originally developed. It would have been helpful if the authors had reported their data associated with the factor structure of the instrument. Information about the factor structure would assist readers to judge the value of calculating means of the subscales. It is valuable for readers to know whether or not the factor structure from the original Solomon and Rothblum study was replicated, and how that had an impact on any conclusions drawn from the data.

Reviewer Expertise:

NA

No decision status is available

# Reviewer report: Reviewer response for version 1

**DOI:** 10.21956/mep.19456.r28669

**Published:** 2017-03-19

**Authors:** Richard Hays

**Affiliations:** 1 James Cook University

This review has been migrated. The reviewer awarded 2 stars out of 5 While I also thought that this would be an interesting contribution, by the end I was not sure what was being measured under the label 'procrastination'. The original reference and tool dated from the 1980's and this does not appear to feature much elsewhere. There is a real risk in relying on something that may not be clear or valid in the context of medical education, although perhaps others have just missed it. So, what is that this tool really measures? How does it relate to measures of personality, resilience and learning preferences? Certainly some medical students are not well engaged, find university work very challenging (often not related to academic ability) and even lack insight - these are the current issues that appear in the literature. Lecture attendance is another issue - lack of organisation, a preference for accessing on-line materials etc may contribute to poor lecture attendance. Also as a single institution study there are limitations, although one other school found similar results. Finally, I am not sure what a training program to overcome procrastination would look like!

Reviewer Expertise:

NA

No decision status is available

# Reviewer report: Reviewer response for version 1

**DOI:** 10.21956/mep.19456.r28671

**Published:** 2017-03-17

**Authors:** John Cookson

**Affiliations:** 1 University of Worcester

This review has been migrated. The reviewer awarded 4 stars out of 5 I found this an interesting study because it looks at student engagement from a different perspective; that of procrastination. It is, as the authors acknowledge, dependent on students accurately recording their views but the completion rate was commendably high. There are other ways of measuring engagement; one of the simplest and best is attendance. It would be interesting to know if there are any correlations. Even so, these measurements are probably all just symptoms rather than the disease itself. Those studying engagement may find the Procrastination Assessment Scale for Students of interest.

Reviewer Expertise:

NA

No decision status is available
